# MeCP2 Promotes Colorectal Cancer Metastasis by Modulating ZEB1 Transcription

**DOI:** 10.3390/cancers12030758

**Published:** 2020-03-23

**Authors:** Dan Luo, Wei Ge

**Affiliations:** National Key Laboratory of Medical Molecular Biology & Department of Immunology, Institute of Basic Medical Sciences, Chinese Academy of Medical Sciences, 5 Dong Dan San Tiao, Dongcheng District, Beijing 100005, China

**Keywords:** colorectal cancer, metastasis, MeCP2, ZEB1, SPI1

## Abstract

*Background*: Recurrence and distant organ metastasis is a major cause of death in colorectal cancer (CRC); however, the underlying molecular mechanisms regulating this phenomenon are poorly understood. MeCP2 is a key epigenetic regulator and is amplified in many types of cancer. Its role in CRC and the molecular mechanisms underlying its action remain unknown. *Methods*: We used western blot and immunohistochemistry to detect MeCP2 expression in CRC tissues, and then investigated its biological functions in vitro and in vivo. Chromatin immunoprecipitation, co-immunoprecipitation, and electrophoretic mobility shift assays were used to detect the associations among MeCP2 (Methyl-CpG binding protein 2), SPI1 (Spi-1 Proto-Oncogene), and ZEB1 (Zinc Finger E-Box Binding Homeobox 1). *Results*: Using the Cancer Genome Atlas and Oncomine databases, we found MeCP2 expression was upregulated in CRC tissues and this upregulation was related to poor prognosis. Meanwhile, MeCP2 depletion (KO/KD) in CRC cells significantly inhibited stem cell frequency, and invasion and migration ability in vitro, and suppressed CRC metastasis in vivo. Mechanistically, we show MeCP2 binds to the transcription factor SPI1, and aids its recruitment to the ZEB1 promoter. SPI1 then facilitates ZEB1 expression at the transcription level. In turn, ZEB1 induces the expression of MMP14, CD133, and SOX2, thereby maintaining CRC stemness and metastasis. *Conclusions*: MeCP2 is a novel regulator of CRC metastasis. MeCP2 suppression may be a promising therapeutic strategy in CRC.

## 1. Introduction

Colorectal cancer (CRC) is the third most common cancer worldwide and the fourth most common cause of cancer death [1]. Although treatment of CRC has improved significantly in the past several decades, mortality was ~9.2% in 2018 [2]. This is mainly ascribed to recurrence and distant organ metastasis [3]. Therefore, understanding the molecular mechanisms driving CRC initiation and metastasis is crucial for CRC treatment.

Methyl-CpG binding protein 2 (MeCP2), a member of the methyl-CpG-binding domain (MBD) family [4], is amplified in a significant proportion of human malignancies [5]. It is a key regulator of gene expression that controls chromatin organization and gene transcription by binding to methylated DNA or gene promoters [6,7]. MeCP2 has a transcriptional repression domain (TRD) and attracts histone deacetylase complexes, thus maintaining local chromatin in a less active state [8]. MeCP2 was also recently shown to recruit transcriptional activators such as CREB1 to the promoter region [9,10,11]. 

The role of MeCP2 in neuronal systems has been well studied: loss of function mutations in the *MECP2* gene have been shown to cause Rett syndrome, a severe neurological disorder in females [12], and duplication of *MECP2* was found to result in autistic-like behaviors [13]. MeCP2 has been well studied in neuronal systems, whereas its role in malignancy is not well-defined. MeCP2 expression is upregulated in lung and breast cancer, and MeCP2 has been shown to promote cancer cell proliferation [14,15]; however, its precise role in malignancy remains unclear. Moreover, the biological characteristics of MeCP2 in CRC is yet to be determined.

In this study, we examined the expression of MeCP2 in CRC patients and its role in facilitating CRC progression. We found MeCP2 depletion repressed the epithelial to mesenchymal transition (EMT) process of CRC cells by inhibiting the expression of the Zinc Finger E-Box Binding Homeobox 1 (ZEB1). Through chromatin immunoprecipitation (CHIP-qPCR), we found that MeCP2 binds to the promoter region of ZEB1 in order to regulate the expression of ZEB1. Through the electrophoretic mobility shift assay (EMSA) and the bio-layer interferometry (BLI) assay, the result showed that the SPI1 protein binds to the promoter region of ZEB1, so we demonstrated a transcription factor, SPI1, which can regulate ZEB1. In particular, we revealed that MeCP2 has a solid interaction with SPI1. Our results provide important insights into the functional mechanism of MeCP2 involved in the progression of CRC.

## 2. Results

### 2.1. MeCP2 is Overexpressed in CRC and is Associated with A Poor Prognosis

**Figure 1:**
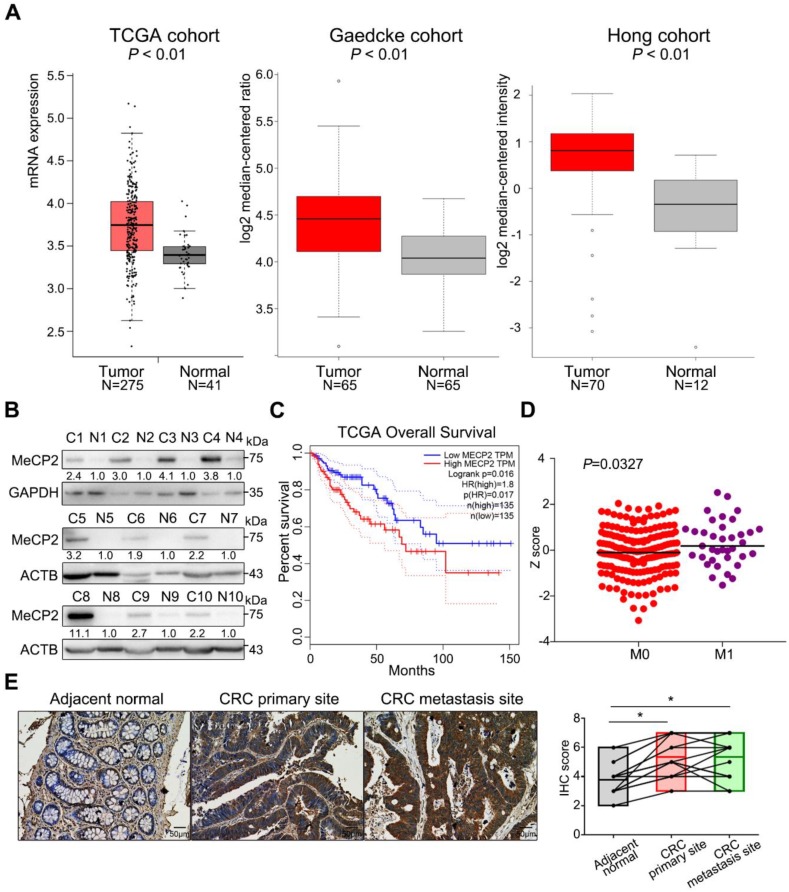
MeCP2 is overexpressed in metastatic colorectal cancer (CRC). (**A**) MeCP2 mRNA is upregulated in CRC tissues compared to normal tissues in the Cancer Genome Atlas (TCGA) cohort (left), and the Oncomine (Gaedcke and Hong) cohorts. (**B**) MeCP2 protein expression is higher in CRC tissues compared to adjacent normal tissues, as determined by Western blot. (**C**) Correlation between MeCP2 expression and survival in TCGA database for CRC, analyzed with the Gene Expression Profiling Interactive Analysis (GEPIA) online analysis tool (Kaplan-–Meier analysis of Overall Survival time). HR, hazard ratio. (**D**) *MECP2* mRNA expression is higher in M1 stage CRC patients compared to M0 stage CRC patients. (**E**) MeCP2 immunohistochemistry (IHC) staining scores in paired adjacent normal, CRC tumor tissues, and liver metastasis tissues (*n* = 15).

To evaluate MeCP2 expression in CRC, we analyzed the TCGA and Oncomine databases [16,17]. The mRNA expression of *MECP2* was significantly higher in CRC tissues than normal tissues (Figure 1A). MeCP2 protein was also upregulated in CRC tissues compared to their adjacent normal tissues, as shown by Western blot (Figure 1B, Table S1 showed patients’ information). We validated the prognostic significance of MeCP2 in the TCGA cohort using the Gene Expression Profiling Interactive Analysis (GEPIA) online analysis tool [18], and found MeCP2 overexpression predicted shorter overall survival (Figure 1C). Next, we examined whether MeCP2 expression was related to CRC metastasis. A significant difference in MeCP2 expression was observed between M1 and M0 stage CRC patients in the TCGA database (Figure 1D). Moreover, immunohistochemical (IHC) staining of paired adjacent normal tissue, primary CRC, and liver metastatic foci revealed MeCP2 overexpression at the protein level (*N* = 15, *p* < 0.05; Figure 1E).

### 2.2. MeCP2 Depletion does not Affect the Proliferation of CRC Cells

To evaluate the functional importance of MeCP2 in CRC cell lines, we constructed artificial shRNAs to generate a stable HCT116 clone, and also used a CRISPR/Cas9 system targeting MeCP2. MeCP2 knockdown or knockout was confirmed by Western blot.

**Figure 2:**
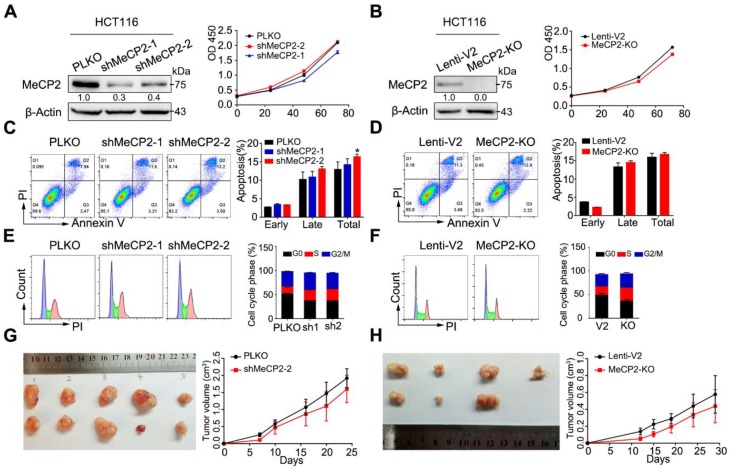
MeCP2 depletion does not influence the proliferation of colorectal cancer (CRC) cells in vitro and in vivo. (**A**) shRNA-mediated MeCP2 knockdown was determined by Western blot (left). MeCP2 silencing did not influence cell viability in HCT116 cells (right). (**B**) sgRNA-mediated MeCP2 knockout was determined by Western blot (left). MeCP2 knockout did not influence cell viability in HCT116 cells (right). (**C**) MeCP2 knockdown did not affect apoptosis of HCT116 cells. (**D**) MeCP2 knockout did not influence apoptosis in HCT116 cells. (**E**) MeCP2 knockdown did not influence the cell cycle distribution. (**F**) MeCP2 knockout did not affect cell cycle distribution. (**G**) MeCP2 knockdown did not suppress cell proliferation in vivo. (**H**) MeCP2 knockout did not influence cell proliferation in vivo.

Knockdown or knockout of MeCP2 did not affect cell growth (Figure 2A,B, Figure S1A,B). In addition, MeCP2 depletion did not induce apoptosis in HCT116, HT29, or SW480 cell lines (Figure 2C,D, Figure S1C,D), and had little effect on the cell cycle (Figure 2E,F, Figure S1E,F). Furthermore, we found MeCP2 depletion in HCT116 cells did not suppress tumor growth in vivo (Figure 2G,H).

### 2.3. Abrogation of MeCP2 Inhibits CRC Cell Migration, Invasion and Stemness

**Figure 3:**
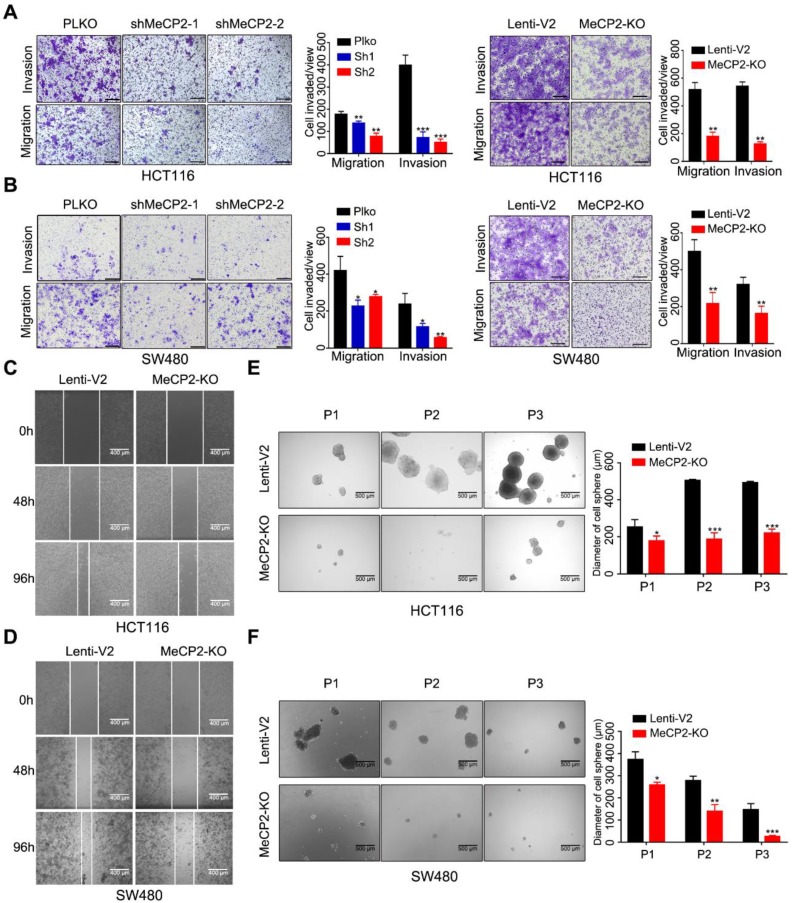
Abrogation of MeCP2 inhibits colorectal cancer (CRC) cell migration, invasion, and stemness. (**A**) MeCP2 knockdown and knockout inhibited HCT116 cell migration and invasion in a Transwell assay. Representative images (left) and quantitative analyses (right) are shown. (**B**) MeCP2 knockdown and knockout inhibited SW480 cell migration and invasion in a Transwell assay. Representative images (left) and quantitative analyses (right) are shown. (**C**) Knockout of MeCP2 in HCT116 cells suppressed migration in a wound-healing assay. Representative images are shown. (**D**) Knockout of MeCP2 in SW480 cells suppressed migration in a wound-healing assay. Representative images are shown. (**E**,**F**) Representative images and quantification of the in vitro sphere-formation assay of MeCP2 knockout in CRC cells and control cells (*n* = 6). P1: Passage 1, P2: Passage 2, P3: Passage 3. Scale bar: 200 µm. * *p* < 0.05, ** *p* < 0.01, *** *p* < 0.001 (Student’s *t*-test).

We next examined whether MeCP2 affects the migratory and invasive properties of CRC cells in vitro. Transwell assays showed knockdown and knockout of MeCP2 in HCT116 and SW480 cells decreased their migration and invasion abilities (Figure 3A,B). The migration abilities of HCT116 and SW480 cells were also impaired after MeCP2 depletion in wound-healing assays (Figure 3C,D). Moreover, reduced sphere numbers and sizes, as well as decreased stem cell frequency, were observed in MeCP2-knockout HCT116 and SW480 cells (Figure 3E,F). 

### 2.4. MeCP2 Knockout Suppresses Metastasis in Nude Mice

**Figure 4:**
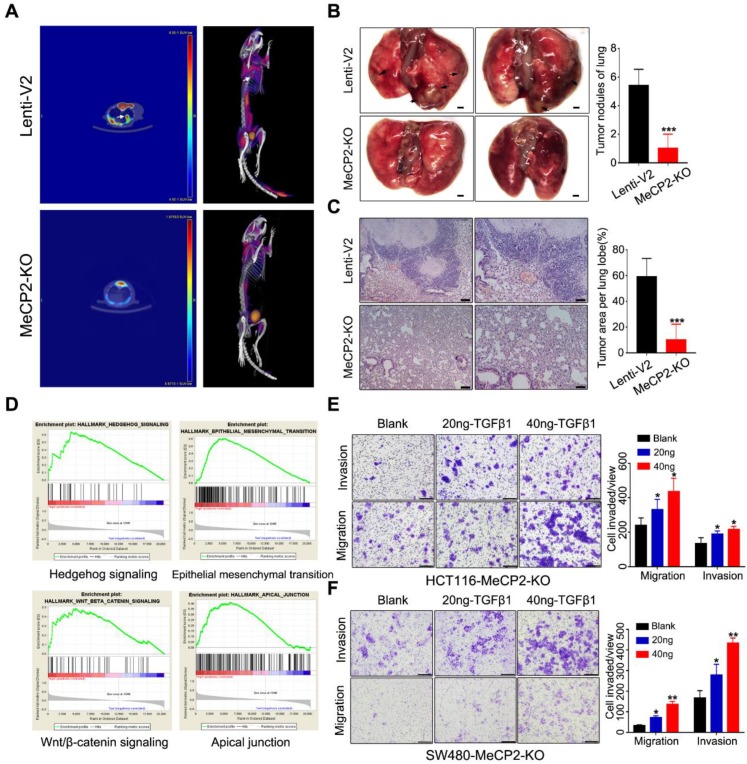
MeCP2 knockout suppresses metastasis in nude mice. (**A**) Comparison of representative transverse PET/CT images of ^18^F-FDG in the tail vein injection of MeCP2-knockout or control HCT116 cells (*n* = 6 mice per group). Arrow: metastatic lung nodules. (**B**) Pictures of metastatic lung nodules in nude mice. The arrows indicate the metastatic tumor on the surface of the lung (left). The number of nodules was quantified (right). (**C**) H&E staining was performed on serial sections of the lung tissue (left). The area containing nodules was quantified (right). (**D**) Representative Gene Set Enrichment Analysis (GSEA) plots enriched in the Cancer Genome Atlas (TCGA) colorectal cancer (CRC) group of high MeCP2 expression, with the significance defined by q < 0.25. (**E**) TGF-β1 treatment abrogated the inhibition of the migration and invasion enforced by MeCP2-knockout in HCT116 cells. (**F**) TGF-β1 treatment abrogated the inhibition of the migration and invasion enforced by MeCP2-knockout in SW480 cells.

To further investigate the effects of MeCP2 on CRC cell invasion and metastasis, we performed metastasis assays in vivo. MeCP2-knockout HCT116 cells and control cells were injected into 6-week-old nude mice via tail-vein injection. Six weeks after injection, we imaged the mice using positron emission tomography/computed tomography (PET/CT) after uptake 18F-FDG (Figure 4A). Eight weeks after injection, we sacrificed the mice and counted the metastasis nodules that formed on the surface of the lungs (Figure 4B). We used H&E staining to detect metastatic lesions on the surfaces of the mouse lungs (Figure 4C). Mice in the control group had more and larger lung metastatic nodules than those injected with MeCP2-knockout HCT116 cells.

To investigate the underlying mechanisms for the role of MeCP2 in CRC metastasis, we performed GSEA of TCGA data based on the MeCP2 expression level in cancer tissues obtained from CRC patients (Table S4). We found a coordinated upregulation of genes implicated in Hedgehog signaling, EMT, Wnt/β-catenin signaling, and the apical junction (Figure 4D). EMT is a process by which epithelial cells lose their cell polarity and cell-cell adhesion, and gain migratory and invasive properties [19]. Throughout tumor progression, extracellular molecules in the tumor microenvironment (TGFβ, Wnt, Hedgehog, etc.) are known to induce EMT, triggering the dissociation of malignant cells from primary tumors [20]. Indeed, we found TGFβ restored the invasion and migration ability of MeCP2-depleted CRC cells (Figure 4E,F). Therefore, EMT-related regulators may be involved in the effect of MeCP2 on tumor cell migration and invasion.

### 2.5. ZEB1 is the Major Effector of MeCP2

**Figure 5:**
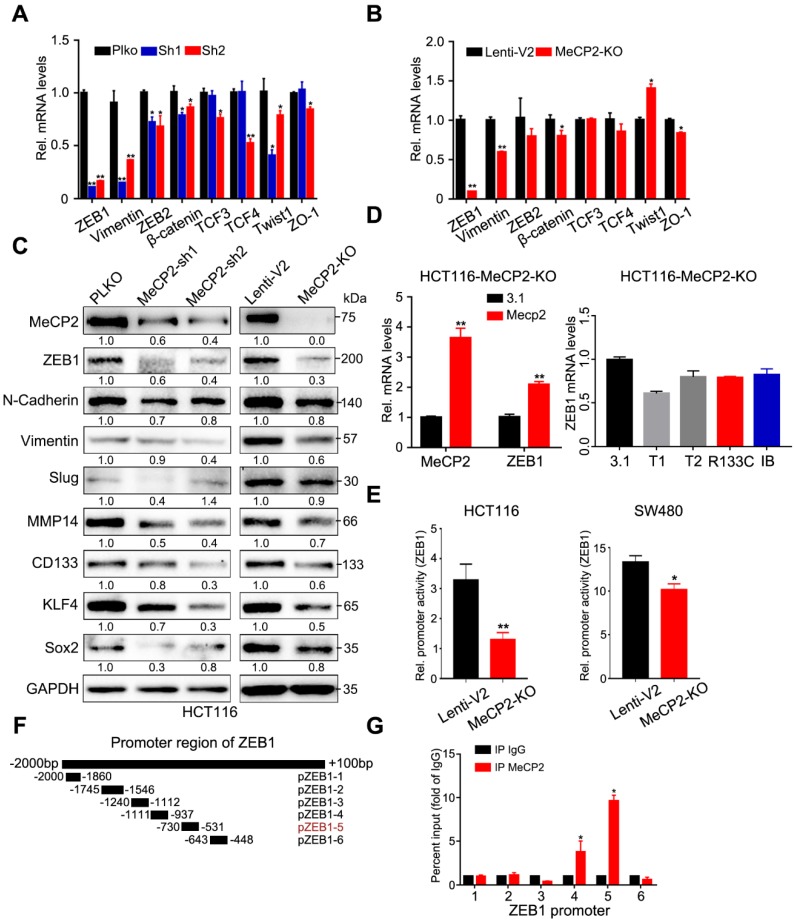
ZEB1 is the major effector of MeCP2. (**A**) The mRNA levels of eight well-known epithelial to mesenchymal transition (EMT)-related genes in HCT116 cells with MeCP2 shRNA were examined by real-time PCR. (**B**) The mRNA levels of eight well-known EMT-related genes in HCT116 cells with MeCP2 sgRNA were examined by real-time PCR. (**C**) Western blot of EMT-related proteins in MeCP2-depleted cells. (**D**) The mRNA levels of *MECP2* and *ZEB1* were examined in MeCP2-knockout (KO) HCT116 cells after MeCP2 overexpression (left). The mRNA levels of *ZEB1* were examined in MeCP2-knockout (KO) HCT116 cells after transfection with different truncation or mutation variants of MeCP2. (**E**) *ZEB1* promoter activity. (**F**) Identification of the MeCP2 binding region on the *ZEB1* promoter using deletion mutants. (**G**) HCT116 cells were subjected to chromatin immunoprecipitation using antibodies against MeCP2 and IgG.

We examined the expression of eight well-known EMT-related genes and found MeCP2 depletion inhibited *ZEB1* expression at the mRNA level (Figure 5A,B). We also showed that MeCP2 abrogation suppressed the expression of N-Cadherin, Vimentin, and Slug proteins via Western blot. The expression of a downstream target gene of ZEB1, MMP14 (a matrix metalloprotease), was also consistently suppressed in MeCP2-depleted HCT116 cells (Figure 5C). Moreover, the expression of cancer stem cell surface antigens, including CD133 (Prominin-Like Protein 1) [21], KLF4 (Kruppel-like factor 4) [22], and Sox2 (SRY-Related HMG-Box Gene 2) [23], was reduced after MeCP2 inhibition (Figure 5C), consistent with the aforementioned observations in the sphere formation assay. Expression of full-length MeCP2 restored ZEB1 mRNA expression in MeCP2-depleted cells (Figure 5D left), but truncated or mutated versions of MeCP2 (Table S5, the sequence of wild type or depletion MeCP2 CDS) had no effect (Figure 5D right).

ZEB1 is a transcription factor that can be regulated at multiple levels, including transcription, mRNA decay, and protein degradation. We found MeCP2 depletion influenced the *ZEB1* promoter activity (Figure 5E), suggesting *ZEB1* is a target gene of MeCP2. In addition, we showed a 200-bp sequence of the *ZEB1* promoter region (pZEB1-5) was the MeCP2-binding site using CHIP assays (Figure 5F,G). 

### 2.6. ZEB1 is A Transcriptional Target of the MeCP2-SPI1 Complex

We next investigated how MeCP2 regulates *ZEB1* mRNA expression. MeCP2 was previously shown to associate with a transcriptional activator at the promoter of an activated target [9]. First, we predicted transcription factors that bind to pZEB1-5 using the UCSC genome browser (Figure S2A). We then used the GEPIA online analysis tool to analyze the correlation between predicated transcription factors and ZEB1 expression in the TCGA database for colon adenocarcinoma (COAD).

**Figure 6:**
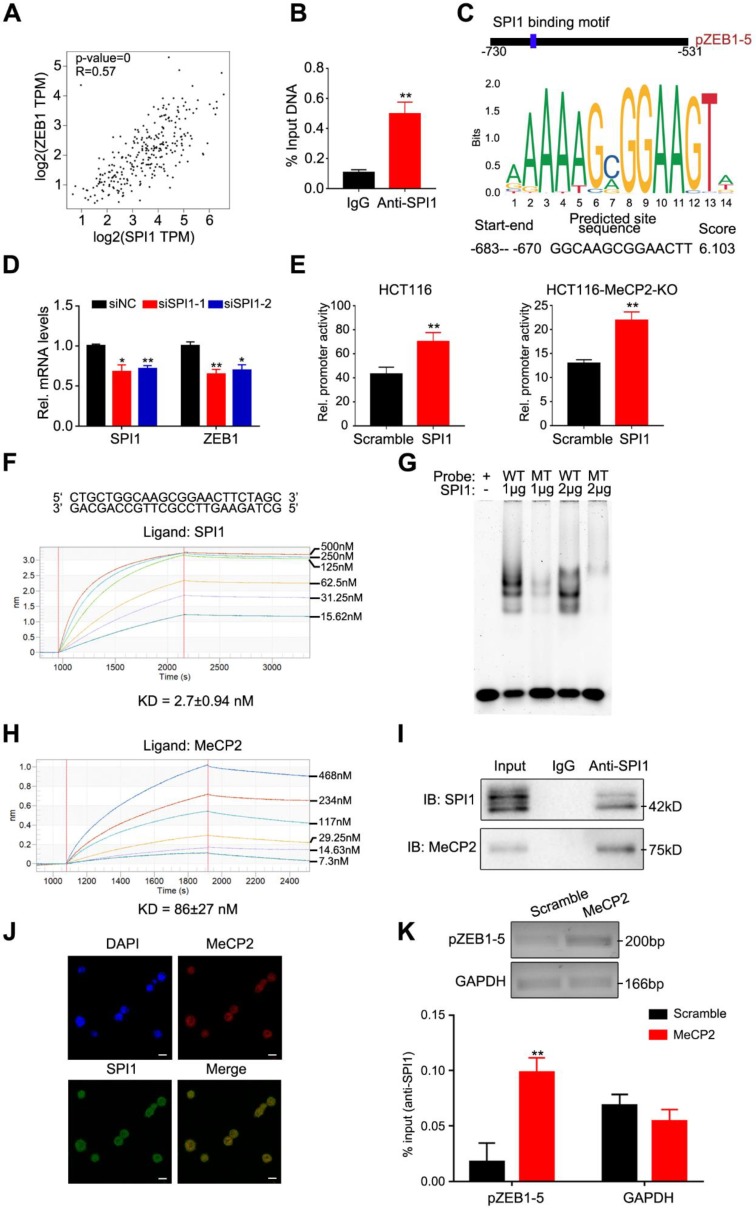
ZEB1 is a transcriptional target of the MeCP2-SPI1 complex. (**A**) Correlation between ZEB1 and SPI1 expression in the Cancer Genome Atlas (TCGA) database for colon adenocarcinoma (COAD), analyzed with the Gene Expression Profiling Interactive Analysis (GEPIA) online analysis tool. (**B**) Real-time PCR analysis of the chromatin immunoprecipitation (CHIP) assay in HCT116-SPI1 cells showing direct binding between the pZEB1-5 region and the SPI1 protein. (**C**) Predicted SPI1 binding sequence in the pZEB1-5 region from the JASPAR website. (**D**) Real-time PCR analysis of ZEB1 regulation by SPI1, with or without SPI1 knockdown. (**E**) Luciferase activity determined after SPI1 overexpression in HCT116 and HCT116-MeCP2-knockout (KO) cells. (**F**) Bio-Layer Interferometry (BLI) analysis of biotin-probe binding to the SPI1 protein. (**G**) Electrophoretic mobility shift assay (EMSA) analysis of the SPI1 protein with the wild type (WT)-probe or the point mutated (MT)-probe. (**H**) BLI analysis of SPI1 interaction with MeCP2. (**I**) Co-immunoprecipitation of SPI1 and MeCP2 in HCT116 cells. (**J**) Co-localization of SPI1 with MeCP2 analyzed by confocal microscopy. (**K**) CHIP-qPCR analysis of SPI1 occupancy at the pZEB1-5 promoter in HCT116 cells transfected with Scramble and MeCP2.

We observed a positive correlation between SPI1 and ZEB1 expression (Figure 6A), and verified that SPI1 predominantly bound to the pZEB1-5 region using CHIP-qPCR (Figure 6B). We predicted that SPI1 binding to ZEB1 utilizes a GGCAAGCGGAACTT motif (Figure 6C). We then used this motif to construct seven biotin-labeled probes to assess MeCP2 binding. We found no obvious difference in the binding affinities of the MBD domain of MeCP2 and biotin-probes via bio-layer interferometry (BLI) analysis (Figure S2B), suggesting MeCP2 has no preference for a specific nucleotide sequence. To confirm whether the pZEB1-5 region was commonly methylated, bisulfite pyrosequencing analysis was performed to examine the methylation levels in HCT116, SW480, normal (*n* = 2) and CRC (*n* = 2) tissues. The CpG islands and the pZEB1-5 region for bisulfite pyrosequencing in the ZEB1 promoter region are shown in Figure S3A. The analysis of pyrosequencing demonstrated that the pZEB1-5 region methylation levels were low (Figure S3B,C). It therefore appears that MeCP2 binds to the *ZEB1* promoter independent of DNA methylation.

Next, we found that the disruption of SPI1 inhibited ZEB1 mRNA expression in HCT116 cells (Figure 6D). Using dual-luciferase assays, we confirmed that the promoter activity of ZEB1 significantly increased after SPI1 overexpression in HCT116 and MeCP2-knockout HCT116 cells (Figure 6E). BLI analysis validated that the SPI1 protein bound to the GGCAAGCGGAACTT motif (Figure 6F), and our electrophoretic mobility shift assay (EMSA) revealed similar data (Figure 6G).

We then determined whether the MeCP2-mediated regulation of *ZEB1* expression relies on SPI1. We found that the SPI1 protein interacted with MeCP2 protein in vitro via BLI analysis (Figure 6H), and confirmed the interaction by co-immunoprecipitation in HCT116 cells (Figure 6I). Confocal analysis of HCT116 cells also indicated that MeCP2 and SPI1 can co-localize (Figure 6J). Furthermore, we found MeCP2 overexpression increased SPI1 occupancy at the *ZEB1* promoter using a CHIP-qPCR assay (Figure 6K). Taken together, our results indicate that *ZEB1* is a transcriptional target of the MeCP2-SPI1 complex.

### 2.7. ZEB1 Restores MeCP2-Mediated Metastasis in MeCP2-Depleted CRC Cells

**Figure 7:**
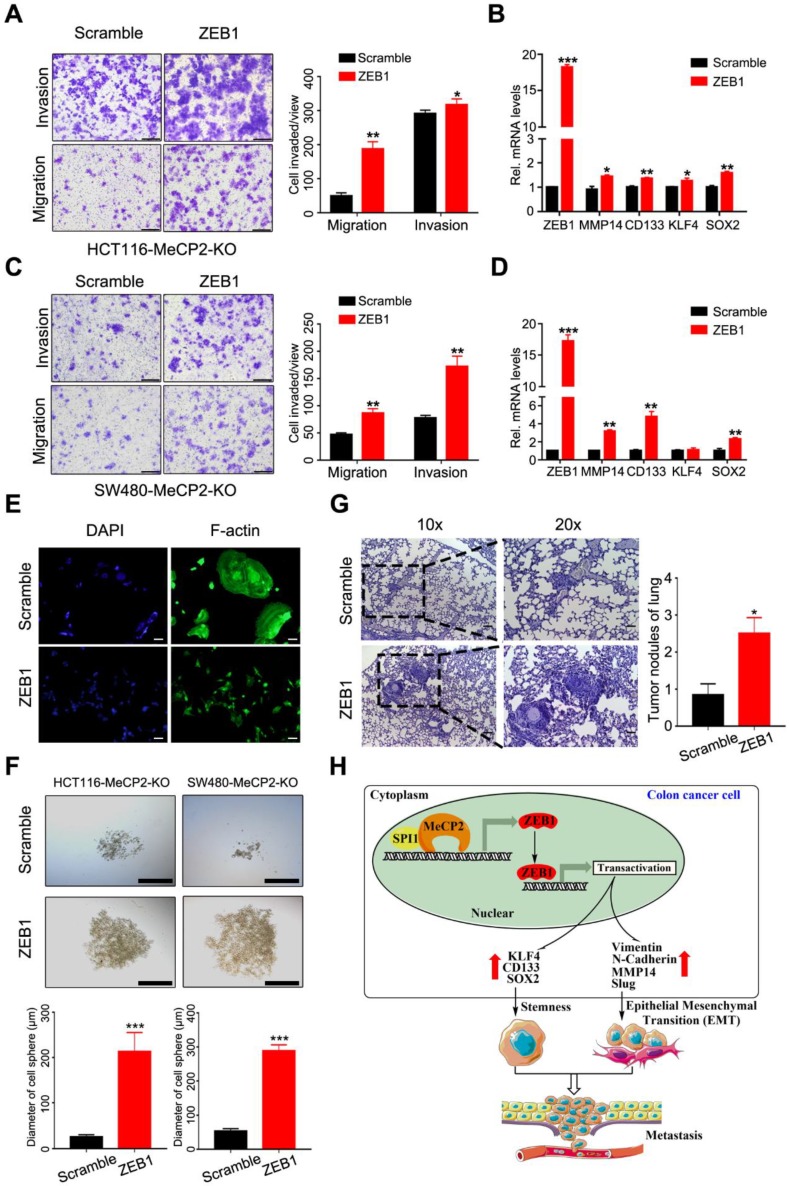
ZEB1 restores the migration, invasion, and metastasis of colorectal cancer cells, and was abrogated by MeCP2 depletion. (**A**) The migration and invasion ability of MeCP2-depleted HCT116 cells was rescued by ZEB1. (**B**) After MeCP2-depleted HCT116 cells were rescued with Scramble or ZEB1, the mRNA levels of *ZEB1*, *MMP14*, *CD133*, *KLF4*, and *SOX2* were examined. (**C**) The migration and invasion ability of MeCP2-depleted SW480 cells was rescued by ZEB1. (**D**) After MeCP2-depleted SW480 cells were rescued with Scramble or ZEB1, the mRNA levels of *ZEB1*, *MMP14*, *CD133*, *KLF4*, and *SOX2* were examined. (**E**) MeCP2-depleted HCT116 cells rescued with Scramble or ZEB1. F-actin was detected with FITC-phalloidin (green). (**F**) Representative images (up) and quantification (low) of the in vitro sphere-formation assay of HCT116-MeCP2-knock out (KO) and SW480-MeCP2-KO rescued with Scramble or ZEB1. (**G**) HCT116-MeCP2-KO cells rescued with Scramble or ZEB1 were injected into nude mice by tail-vein injection (*n* = 8 mice/group). H&E staining was performed on serial sections of the lung tissue (left). Quantification of the number of lung metastatic nodules is shown. (**H**) Schematic diagram showing the proposed mechanism of action of MeCP2 in CRC.

We next investigated whether ZEB1 could abrogate the inhibition of metastasis by ectopically expressing ZEB1 in MeCP2-depleted CRC cells. We found ZEB1 overexpression rescued the inhibition of invasion and migration enforced by MeCP2 depletion in CRC cells (Figure 7A,C). Moreover, ZEB1 overexpression increased the mRNA expression levels of *MMP14*, *CD133*, and *SOX2* (Figure 7B,D). ZEB1 overexpression also resulted in cell morphology changes, including cell elongation and membrane protrusions (Figure 7E). In addition, ZEB1 overexpression restored the stemness of MeCP2-depleted CRC cells (Figure 7F) and impaired the inhibition of lung metastasis induced by MeCP2 depletion (Figure 7G). Moreover, analysis of TCGA Colon Adenoma using the GEPIA tool indicated the prognostic significance of ZEB1 and MMP14; we found ZEB1 and MMP14 upregulation predicted shorter disease free survival, but had no significant effect on overall survival (Figure S4A,B).

As illustrated schematically in Figure 7H, we found that MeCP2 contributes to an increase in the transcription of ZEB1. Mechanically, we propose that MeCP2 interacts with SPI1, then binds to the promoter region of ZEB1 to drive *ZEB1* expression. In turn, ZEB1 expression promotes CRC cell stemness and metastasis through its downstream target genes (including MMP14, CD133, and SOX2), leading to CRC progression.

## 3. Discussion

As a key regulator of gene expression, MeCP2 has been studied extensively with regard to Rett syndrome (RTT), a severe neurological disorder in females [24]. A total of 85–90% of RTT cases result from MeCP2 mutations [25]. Missense mutations that cause RTT are concentrated in two discrete clusters: the Methyl-CpG Binding Domain (MBD) and the NCoR/SMRT Interaction Domain (NID) [26,27]. In this study, we tested whether the truncated/mutant MeCP2 proteins retained the ability to regulate ZEB1, whereas this ability was abolished in the T1 and T2 truncation, R133C mutation, Isoform B. It therefore appears that full-length MeCP2 is required for regulating ZEB1 expression. 

There is growing evidence that MeCP2 is frequently amplified and overexpressed in human cancers [5]. Recently, it was reported that knockdown of MeCP2 repressed the proliferation of tumor cells [28,29]. Zhao et al. found that MeCP2 promotes gastric cancer cell proliferation via the FOXF1-mediated Wnt5a/β-Catenin pathway and suppresses apoptosis through the MYOD1-mediated Caspase-3 pathway [30]. In hepatocellular carcinoma, MeCP2 promotes the proliferation of human HCC HepG2 cells with activation of ERK1/2 signaling pathways [14]. However, in our study, we showed that MeCP2 is upregulated in CRC tissues and is associated with poor survival. In our experiments, despite significant decreases in the size of some tumors, there was no statistically significant difference in the results. We performed CCK-8, cell apoptosis, and cell cycle assays in CRC cell lines. Although some data revealed that MeCP2-depletion had a weak effect on cell proliferation, on the whole, we believe that knockdown or knockout of MeCP2 has no significant inhibitory effect on cell proliferation. In addition, the results of tumor formation in nude mice were influenced by a variety of limiting factors, such as regional growth differences and regional vascular supply differences in nude mice. We found that MeCP2 depletion significantly suppressed the invasion and migration of CRC cells, which suggests that MeCP2 may promote CRC metastasis.

EMT is associated with the initiation of metastasis during cancer progression [31]. We found that MeCP2 depletion inhibited expression of the EMT-inducing transcription factor, ZEB1. Moreover, MeCP2 depletion abolished the expression of downstream target genes of ZEB1, including MMP14. ZEB1 was previously shown to enhance MMP14 expression, thereby promoting the remodeling of the basement membrane and fostering invasion into surrounding tissues [32]. We speculate that MeCP2 regulates the ZEB1/MMP14 axis, and thus affects the migration and invasion of tumor cells. We also showed that MeCP2 depletion significantly reduced the stem cell frequency in CRC. Indeed, ZEB1 was previously reported to regulate stemness by targeting stemness-associated factors like Sox2 and KLF4 [33], suggesting ZEB1 is the major effector of MeCP2 in promoting CRC stemness.

MeCP2 was previously shown to interact with a transcriptional activator occupying the promoter of an activated target gene [9]. We performed a combination of CHIP-qPCR and transcription factor prediction and identified *ZEB1* as a SPI1-targeting gene. We confirmed that MeCP2 binds to SPI1 at the promoter of *ZEB1*. Bian and colleagues reported their work in which they showed that MeCP2 recruited H3K9 to the promoter of miR-200c by interacting with SUV39H1 regulation EMT in glioma cells [34]. Our work suggests MeCP2 uses a different molecular mechanism to drive tumor progression in CRC: MeCP2 binds to SPI1 and aids its recruitment to the *ZEB1* promoter, which in turn, affects *ZEB1* expression at the transcription level.

Pioneering work from the Bird lab showed MeCP2 preferentially binds methylated DNA without any sequence specificity [35], and determined the minimal region (MBD) required to bind DNA, which was ~85 amino acids in length [26]. Here we showed the MBD also binds to non-methylated DNA sequences, without any obvious sequence specificity. As the bisulfite pyrosequencing analysis of the ZEB1 promoter region in HCT116 and SW480 showed the methylation levels of pZEB1-5 were low, it is likely that MeCP2 binds to its target DNA independent of methylation modifications. 

## 4. Materials and Methods

### 4.1. Patients and Tumor Tissue Specimens

A total of 10 formaldehyde-fixed paraffin-embedded CRC specimens and paired adjacent normal tissue samples, were obtained from the Peking Union Medical College Hospital (Beijing, China). The clinical characteristics of the samples are summarized in Table S1. The study was performed with the approval of the Ethics Committee of Peking Union Medical College Hospital. All animal experiments were performed in accordance with a protocol approved by the ethics committee of Institute of Basic Medical Sciences, China.

A constructed tissue microarray containing 15 CRC tissues, with paired adjacent normal tissues and matched liver metastasis tissues, was obtained from the Shanghai Outdo Biotech Corporation (Shanghai, China). Clinicopathological data is shown in Table S2. 

The study was performed with the approval of the Ethics Committee of Peking Union Medical College Hospital. All animal experiments were performed in accordance with a protocol approved by the ethics committee of Institute of Basic Medical Sciences, China.

### 4.2. Lentiviral Infection, Establishment of Stable Cell Lines, and CRISPR Generation

Stable cell lines were established with a lentiviral vector. Briefly, lentivirus was produced by the co-transfection of HEK293T cells with lentivirus expression vectors and the lentiviral packaging plasmid mix (Gag-Pol, Rev, VSVG) according to the standard protocol. Transfection was performed with Lipofectamine 3000 (Invitrogen). The lentiviruses packaging MECP2 shRNA were purchased from LQbiotech (Shanghai, China). To establish stable MECP2 knockdown cell lines, CRC cells were transduced with lentiviruses and then selected with puromycin (2 µg/ml, S7417, Selleck) after 2 days production. 

For the ZEB1 rescue experiment, ZEB1-CDS (ZEB1 coding sequence) were cloned into the pLVX vector with a FLAG tag, and then the empty vector or the ZEB1-CDS were transduced into the MECP2 knockout CRC cells. Guide-RNA was cloned into lentiCRISPRv2. Cells were subsequently infected with lentiviruses, then selected with puromycin as described above for 7 days. The puro+ cells were limiting diluted into 96-well plates. Cells were incubated at 37 ℃ in a CO_2_ incubator for 2–3 weeks for single-clone generation.

### 4.3. Chromatin immunoprecipitation (CHIP)-qPCR

CHIP was conducted according to the manufacturer’s protocol (Active Motif, Carlsbad, USA). Briefly, HCT116 cells were cross-linked with 1% formaldehyde at room temperature for 15 min, and the reaction stopped by 0.125 M glycine addition. Cross-linked cells were then scraped for pellet and resuspended in 500 μL lysis buffer with 1X protease inhibitor. Nuclear lysates were sonicated using a sonicator (QSONICA, Q800R, Newtown USA). Chromatin was sonicated into fragments of approximately 200 bp in size, the supernatants were precleared with agarose beads for 1 hour at 4 °C and then the supernatants were incubated with 10 µg MeCP2 antibody (CST, #3456), SPI1 antibody (CST, #2258) or rabbit IgG as a negative control overnight at 4 °C. DNA–protein complexes were captured using Protein G agarose and eluted in TE buffer at 37 °C. After reverse-crosslinking, the DNA was analyzed via qRT-PCR using specific primers. (Table S3) 

### 4.4. Electrophoretic Mobility Shift Assay (EMSA)

EMSA was performed using an EMSA kit (Invitrogen, Carlsbad, E33075, USA) according to the manufacturer′s instructions. Oligonucleotides representing the wild type (WT) ZEB1 promoter sequence (forward: 5′ CTGCTGGCAAGCGGAACTTCTAGC and reverse: 5′ GCTAGAAGTTCCGCTTGC-CAGCAG) or point mutated (MT) sequence (forward: 5′ CTGCTGGCAAGCAAAACTTCTAGC and reverse: 5′ GCTAGAAGTTTTGCTTGC-CAGCAG) were annealed. A double-stranded DNA probe were added to the purified SPI1 protein (OriGene, AR51789PU-N) in the 5X binding buffer which is 750 mM KCl, 0.5 mM EDTA, 50 mM Tris, pH 7.4. The protein–DNA mixture was incubated at room temperature for 20 min before electrophoresis in 6% acrylamide gel. After electrophoresis, the gel was stained with SYBR Green and then was visualized by a laser-based scanner at 488 nm (GE, Typhoon, FLA9500, Fairfield, USA).

### 4.5. Dual Luciferase Assays

The full-length human ZEB1 promoter cDNA (−2000 bp–+100 bp; UCSC hg38 DNA range = chr10:31317125–31319225) was ligated into the promoter-driven luciferase reporter plasmid pGL3-basic. The cells were plated in 24-well plates, then the cells were transfected with pGL3-basic, pRL-TK for 48 hours. A dual-luciferase reporter assay system (Promega, Madison, WI, USA) was used to calculate luciferase activity. The Renilla luciferase gene was used as the internal reference. Relative luciferase acitivity = firefly luciferase activity/renilla luciferase activity.

### 4.6. Bio-Layer Interferometry (BLI) Assays

The kinetics of MeCP2 binding to SPI1 (Spi-1 Proto-Oncogene) and biotin-nucleotide probe binding to SPI1 was measured using an Octet Red96 instrument. All assays were performed with agitation set to 1000 rpm in PBST buffer. The final volume for all the solutions was 200 μL/well. For determining binding of the biotin-nucleotide probe to SPI1, streptavidin sensors were coated with the biotin-nucleotide. For determining MeCP2 binding to SPI1, 20 μg/mL of the His-SPI1 fusion protein was loaded onto the Anti-His sensors. A two-fold concentration gradient of analyte was used in a titration series of six. Octet data were processed by fortéBio’s data acquisition software v.8.1. Global fitting of the data sets was performed to obtain the optimal results. The KD value was determined using the kon and kdis values.

### 4.7. Gene Set Enrichment Analysis (GSEA)

GSEA software v3.0 (http://www.broadinstitute.org/gsea) was used for all analyses, using hallmark gene sets. MECP2 expression values were used as the phenotype. For analysis of The Cancer Genome Atlas (TCGA) dataset, gene expression data was downloaded from the cBioPortal. 

### 4.8. Statistical Analysis

Quantitative data were presented as the mean ± standard deviation (SD) of three independent experiments and analyzed by the Student’s t-test or the ANOVA test. A P-value < 0.05 was considered statistically significant.

All data generated or analyzed during this study are included either in this article or in the Supplementary materials and method.

## 5. Conclusions

Our findings suggest that MeCP2 interacts with SPI1 and delivers it to the ZEB1 promoter, resulting in transcriptional activation of ZEB1, and subsequent upregulation of downstream target genes involved in tumor cell metastasis and stemness. This makes MeCP2 a promising therapeutic target for abrogating the stemness and metastasis of CRC cells. Therefore, identification of a specific inhibitor of MeCP2 would be beneficial for the development of a novel targeted therapy for CRC.

## Supplementary Materials

The following are available online at https://www.mdpi.com/2072-6694/12/3/758/s1, **Table S1.** Information about patients recruited from Peking Union Medical College Hospital; **Table S2.** Information about patients of tissue microarray; **Table S3.** The primer sequence of CHIP-Qpcr; **Table S4.** GSEA analysis of MeCP2 expression in TCGA; **Table S5.** The sequence of wild type or depletion MeCP2 CDS; **Figure S1 A** sgRNA-mediated MeCP2 knockout determined by Western blot (left). MeCP2 abrogation did not influence cell viability in HT29 cells (right). **B** sgRNA-mediated MeCP2 knockout determined by Western blot (left). MeCP2 abrogation did not influence cell viability in SW480 cells (right). **C** MeCP2 knockdown did not affect apoptosis of HT29 cells. **D** MeCP2 knockout did not influence apoptosis in SW480 cells. **E** MeCP2 knockout in HT29 cells did not influence the cell cycle distribution. **F** MeCP2 knockout in SW480 cells did not affect cell cycle distribution; **Figure S2 A** Prediction of transcription factor binding to the pZEB1-5 promoter region using the UCSC genome browser. **B** Bio-Layer Interferometry (BLI) analysis of the binding of seven biotin-nucleotides to the minimal binding domain (MBD) of the MeCP2 protein; **Figure S3 A** Schematic of the CpG islands and bisulfite pyrosequencing region in the ZEB1 promoter. Input sequence: red region; CpG islands: blue region. **B** Bisulfite pyrosequencing analysis of the ZEB1 promoter region in HCT116 and SW480 cells. **C** Bisulfite pyrosequencing analysis of the ZEB1 promoter region in normal (*n* = 2) and CRC (*n* = 2) tissues; **Figure S4 A** Correlation between ZEB1 expression and survival in TCGA database for CRC, Kaplan–Meier analysis of overall survival time (left) and disease free survival time (right). **B** Correlation between MMp14 expression and survival in TCGA database for CRC, Kaplan–Meier analysis of overall survival time (left) and disease free survival time (right).

## Abbreviations

CRC	colorectal cancer
IHC	immunohistochemistry
MeCP2	Methyl-CpG binding protein 2
SPI1	Spi-1 Proto-Oncogene
ZEB1	Zinc Finger E-Box Binding Homeobox 1
MMP14	Matrix Metallopeptidase 14
CD133	Prominin-Like Protein 1
SOX2	SRY-Related HMG-Box Gene 2
