# Supplementary material for: MeCP2 Promotes Colorectal Cancer Metastasis by Modulating ZEB1 Transcription
Source: Cancers (Basel). 2020 Mar 23;12(3):758. doi: 10.3390/cancers12030758 (PMC7140043; doi:10.3390/cancers12030758)
Supplement: Supplementary file 1 [file cancers-12-00758-s001.pdf]

Supplementary Tables

Table S1. Information about patients recruited from Peking Union Medical College Hospital.

Patient ID	Age (years)	Gender	Location	TNM stage	AJCC stage
1	66	female	Colon	T2N1a	III
2	78	male	Rectum	T2N1b	III
3	72	female	Colon	T3N0	II
4	67	female	Colon	T2N0	I
5	61	female	Rectum	T3N2aM0	III
6	76	female	Rectum	T1N0M0	I
7	67	female	Rectum	T3N0M0	II
8	60	female	Rectum	T2N2aM0	III
9	74	female	Rectum	T2N1aM0	III
10	82	male	Colon	T3N1aM0	III

Table S2. Information about patients of tissue microarray.

Patient ID	Age (years)	Gender	Location	Metastatic locationg	Diameter of Tumor (cm)	TNM stage
1	53	female	Colon		5*4*2.5	T4N2aM0
	54			Liver	2.5*2*2	N1bM1
2	NA	female	Colon		4*4*1.5	T3N0M0
	NA			Liver	5*3.5*3	M1
3	61	male	Colon		3.5*3*1	T3N0M0
	62			Liver	3.5*3*2.7	M1
4	66	female	Colon		3*3*1.5	T3N1bM0
	67			Liver	3*2*2	N0M1
5	60	male	Colon		9*7*1	NA
	60			Liver	7.5*6*4.8	M1
6	73	male	Colon		11*7*3	T3N1aM0
	73			Liver	5*4*4	M1
7	61	male	Colon		4*3.5*1.5	T3N0M0
	62			Liver	2*2*1.5	M1
8	50	male	Colon		NA	NA
	51			Liver	5*5*3	M1
9	46	male	Rectum		4*4*1	N1bM0
	48			Liver	11*7*5	M1
10	67	male	Rectum		4*4*2	N2aM0
	69			Liver	4*3*2.5	M1
11	NA	male	Rectum		4*4*1	NA
	NA			Liver	4*3*3	M1
12	59	male	Rectum		4*1.5*1	T3N0
	59			Liver	6.5*4*3.5	M1

13	63	male	Rectum		4*3*1	N1aM0
	63			Liver	NA	M1
14	53	female	Rectum		3*2*0.5	N2aM0
	54			Liver		M1

NA: not availabe

Table S3. The primer sequence of CHIP-qPCR.

**qPCR primers (human)**	
GAPDH-F	5′- TACTAGCGGTTTTACGGGCG-3′
GAPDH-R	5′- TCGAACAGGAGGAGCAGAGAGCGA-3′
p*ZEB1*-1-F	AGGATGAACACTGAAGGAAGC
p*ZEB1*-1-R	ACCAGAAAGGAAAAAAAAACT
p*ZEB1*-2-F	AACTCACACTTAACCAAGC
p*ZEB1*-2-R	GTCCATGAAATGTGACTGT
p*ZEB1*-3-F	GGTCACGTTTCAGTTTTCT
p*ZEB1*-3-R	CGAATAAGGAAACGTCTTT
p*ZEB1*-4-F	AAGGAGGTGGGAAGCAGGA
p*ZEB1*-4-R	CAGATGTGATCTCTGAACC
p*ZEB1*-5-F	AAGCACCGTGTGGGTATT
p*ZEB1*-5-R	CGGAGAGAGGCTACCTGA
p*ZEB1*-6-F	GTTCAATCTCATTGAAGTCACTTCC
p*ZEB1*-6-R	AAGGGATCGCGGTCTGGACTC

F, forward; R, reverse.

Table S4. GSEA analysis of MeCP2 expression in TCGA.

**Name**	**NES**	**FDR q-val**
HALLMARK_HEDGEHOG_SIGNALING	1.93	0.045
HALLMARK_EPITHELIAL_MESENCHYMAL_TRANSITION	1.67	0.241
HALLMARK_WNT_BETA_CATENIN_SIGNALING	1.65	0.185
HALLMARK_MYOGENESIS	1.65	0.140
HALLMARK_UV_RESPONSE_DN	1.58	0.181
HALLMARK_APICAL_JUNCTION	1.54	0.193

Table S5 The sequence of wild type or depletion MeCP2 CDS.

Vectors	Sequence
MeCP2 CDS (wild type)	ATGGTAGCTGGGATGTTAGGGCTCAGGGAAGAAAAGTCAGAAGACC<br>AGGACCTCCAGGGCCTCAAGGACAAACCCCTCAAGTTTAAAAAGGTG<br>AAGAAAGATAAGAAAGAAGAGAAAGAGGGCAAGCATGAGCCCGTG<br>CAGCCATCAGCCCACCACTCTGCTGAGCCCGCAGAGGCAGGCAAAG<br>CAGAGACATCAGAAGGGTCAGGCTCCGCCCCGGCTGTGCCGGAAGCT<br>TCTGCCTCCCCCAAACAGCGGCGCTCCATCATCCGTGACCGGGGACC<br>CATGTATGATGACCCCACCCTGCCTGAAGGCTGGACACGGAAGCTTA<br>AGCAAAGGAAATCTGGCCGCTCTGCTGGGAAGTATGATGTGTATTTG<br>ATCAATCCCCAGGGAAAAGCCTTTCGCTC……AGGAGAGCAGCCCCA<br>AGGGGCGCAGCAGCAGCGCCTCCTCACCCCCCAAGAAGGAGCACCA<br>CCACCATCACCACCACTCAGAGTCCCCAAAGGCCCCCGTGCCACTGC<br>TCCCACCCCTGCCCCCACCTCCACCTGAGCCCGAGAGCTCCGAGGAC<br>CCCACCAGCCCCCCTGAGCCCCAGGACTTGAGCAGCAGCGTCTGCAA<br>AGAGGAGAAGATGCCCAGAGGAGGCTCACTGGAGAGCGACGGCTGC<br>CCCAAGGAGCCAGCTAAGACTCAGCCCGCGGTTGCCACCGCCGCCAC<br>GGCCGCAGAAAAGTACAAACACCGAGGGGAGGGAGAGCGCAAAGA<br>CATTGTTTCATCCTCCATGCCAAGGCCAAACAGAGAGGAGCCTGTGG<br>ACAGCCGGACGCCCGTGACCGAGAGAGTTAGCTGA
MeCP2 truncation CDS (T1)	~~ATGGTAGCTGGGATGTTAGGGCTCAGGGAAGAAAAGTCAGAAGACC~~<br>~~AGGACCTCCAGGGCCTCAAGGACAAACCCCTCAAGTTTAAAAAGGTG~~<br>~~AAGAAAGATAAGAAAGAAGAGAAAGAGGGCAAGCATGAGCCCGTG~~<br>~~CAGCCATCAGCCCACCACTCTGCTGAGCCCGCAGAGGCAGGCAAAG~~<br>~~CAGAGACATCAGAAGGGTCAGGCTCCGCCCCGGCTGTGCCGGAAGCT~~<br>~~TCTGCCTCCCCCAAACAGCGGCGCTCCATCATCCGT~~GACCGGGGACC<br>CATGTATGATGACCCCACCCTGCCTGAAGGCTGGACACGGAAGCTTA<br>AGCAAAGGAAATCTGGCCGCTCTGCTGGGAAGTATGATGTGTATTTG<br>ATCAATCCCCAGGGAAAAGCCTTTCGCTC……AGGAGAGCAGCCCCA<br>AGGGGCGCAGCAGCAGCGCCTCCTCACCCCCCAAGAAGGAGCACCA<br>CCACCATCACCACCACTCAGAGTCCCCAAAGGCCCCCGTGCCACTGC<br>TCCCACCCCTGCCCCCACCTCCACCTGAGCCCGAGAGCTCCGAGGAC<br>CCCACCAGCCCCCCTGAGCCCCAGGACTTGAGCAGCAGCGTCTGCAA<br>AGAGGAGAAGATGCCCAGAGGAGGCTCACTGGAGAGCGACGGCTGC

	CCCAAGGAGCCAGCTAAGACTCAGCCCGCGGTTGCCACCGCCGCCAC<br>GGCCGCAGAAAAGTACAAACACCGAGGGGAGGGAGAGCGCAAAGA<br>CATTGTTTCATCCTCCATGCCAAGGCCAAACAGAGAGGAGCCTGTGG<br>ACAGCCGGACGCCCGTGACCGAGAGAGTTAGCTGA
MeCP2 truncation CDS (T2)	ATGGTAGCTGGGATGTTAGGGCTCAGGGAAGAAAAGTCAGAAGACC<br>AGGACCTCCAGGGCCTCAAGGACAAACCCCTCAAGTTTAAAAAGGTG<br>AAGAAAGATAAGAAAGAAGAGAAAGAGGGCAAGCATGAGCCCGTG<br>CAGCCATCAGCCCACCACTCTGCTGAGCCCGCAGAGGCAGGCAAAG<br>CAGAGACATCAGAAGGGTCAGGCTCCGCCCCGGCTGTGCCGGAAGCT<br>TCTGCCTCCCCCAAACAGCGGCGCTCCATCATCCGTGACCGGGGACC<br>CATGTATGATGACCCCACCCTGCCTGAAGGCTGGACACGGAAGCTTA<br>AGCAAAGGAAATCTGGCCGCTCTGCTGGGAAGTATGATGTGTATTTG<br>ATCAATCCCCAGGGAAAAGCCTTTCGCTC……AGGAGAGCAGCCCCA<br>AGGGGCGCAGCAGCAGCGCCTCCTCACCCCCCAAGAAGGAG~~CACCA~~<br>~~CCACCATCACCACCACTCAGAGTCCCCAAAGGCCCCCGTGCCACTGC~~<br>~~TCCCACCCCTGCCCCCACCTCCACCTGAGCCCGAGAGCTCCGAGGAC~~<br>~~CCCACCAGCCCCCCTGAGCCCCAGGACTTGAGCAGCAGCGTCTGCAA~~<br>~~AGAGGAGAAGATGCCCAGAGGAGGCTCACTGGAGAGCGACGGCTGC~~<br>~~CCCAAGGAGCCAGCTAAGACTCAGCCCGCGGTTGCCACCGCCGCCAC~~<br>~~GGCCGCAGAAAAGTACAAACACCGAGGGGAGGGAGAGCGCAAAGA~~<br>~~CATTGTTTCATCCTCCATGCCAAGGCCAAACAGAGAGGAGCCTGTGG~~<br>~~ACAGCCGGACGCCCGTGACCGAGAGAGTTAGCTGA~~
MeCP2 mutation CDS (R133C)	ATGGTAGCTGGGATGTTAGGGCTCAGGGAAGAAAAGTCAGAAGACC<br>AGGACCTCCAGGGCCTCAAGGACAAACCCCTCAAGTTTAAAAAGGTG<br>AAGAAAGATAAGAAAGAAGAGAAAGAGGGCAAGCATGAGCCCGTG<br>CAGCCATCAGCCCACCACTCTGCTGAGCCCGCAGAGGCAGGCAAAG<br>CAGAGACATCAGAAGGGTCAGGCTCCGCCCCGGCTGTGCCGGAAGCT<br>TCTGCCTCCCCCAAACAGCGGCGCTCCATCATCCGTGACCGGGGACC<br>CATGTATGATGACCCCACCCTGCCTGAAGGCTGGACACGGAAGCTTA<br>AGCAAAGGAAATCTGGCCGCTCTGCTGGGAAGTATGATGTGTATTTG<br>ATCAATCCCCAGGGAAAAGCCTTTUGCTC……AGGAGAGCAGCCCCA<br>AGGGGCGCAGCAGCAGCGCCTCCTCACCCCCCAAGAAGGAGCACCA<br>CCACCATCACCACCACTCAGAGTCCCCAAAGGCCCCCGTGCCACTGC<br>TCCCACCCCTGCCCCCACCTCCACCTGAGCCCGAGAGCTCCGAGGAC<br>CCCACCAGCCCCCCTGAGCCCCAGGACTTGAGCAGCAGCGTCTGCAA<br>AGAGGAGAAGATGCCCAGAGGAGGCTCACTGGAGAGCGACGGCTGC<br>CCCAAGGAGCCAGCTAAGACTCAGCCCGCGGTTGCCACCGCCGCCAC<br>GGCCGCAGAAAAGTACAAACACCGAGGGGAGGGAGAGCGCAAAGA<br>CATTGTTTCATCCTCCATGCCAAGGCCAAACAGAGAGGAGCCTGTGG<br>ACAGCCGGACGCCCGTGACCGAGAGAGTTAGCTGA
MeCP2 isoform CDS (IB)	ATGGCCGCCGCCGCCGCCGCCGCGCCGAGCGGAGGAGGAGGAGGAG<br>GCGAGGAGGAGAGACTGGAAGAAAAGTCAGAAGACCAGGACCTCC<br>AGGGCCTCAAGGACAAACCCCTCAAGTTTAAAAAGGTGAAGAAAGA<br>TAAGAAAGAAGAGAAAGAGGGCAAGCATGAGCCCGTGCAGCCATCA

GCCCACCACTCTGCTGAGCCCGCAGAGGCAGGCAAAGCAGAGACAT
CAGAAGGGTCAGGCTCCGCCCCGGCTGTGCCGGAAGCTTCTGCCTCC
CCCAAACAGCGGCGCTCCATCATCCGTGACCGGGGACCCATGTATGA
TGACCCCACCCTGCCTGAAGGCTGGACACGGAAGCTTAAGCAAAGG
AAATCTGGCCGCTCTGCTGGGAAGTATGATGTGTATTTGATCAATCCC
CAGGGAAAAGCCTTTCGCTC……AGGAGAGCAGCCCCAAGGGGCGC
AGCAGCAGCGCCTCCTCACCCCCCAAGAAGGAGCACCACCACCATC
ACCACCACTCAGAGTCCCCAAAGGCCCCCGTGCCACTGCTCCCACCC
CTGCCCCCACCTCCACCTGAGCCCGAGAGCTCCGAGGACCCCACCAG
CCCCCCTGAGCCCCAGGACTTGAGCAGCAGCGTCTGCAAAGAGGAG
AAGATGCCCAGAGGAGGCTCACTGGAGAGCGACGGCTGCCCCAAGG
AGCCAGCTAAGACTCAGCCCGCGGTTGCCACCGCCGCCACGGCCGCA
GAAAAGTACAAACACCGAGGGGAGGGAGAGCGCAAAGACATTGTTT
CATCCTCCATGCCAAGGCCAAACAGAGAGGAGCCTGTGGACAGCCG
GACGCCCGTGACCGAGAGAGTTAGCTGA

The right parts represent the mutation, the right with Strikethrough represent deletion sequence.

Materials and methods

Cell culture

The CRC cell lines HCT116, SW480, and HT29 were purchased from the National Infrastructure of Cell Line Resource (China, Beijing). HCT116 cells were cultured in IMDM supplemented with 10% FBS. SW480 cells were cultured in DMEM supplemented with 10% FBS. HT29 cells were cultured in DMEM/F12 supplemented with 10% FBS. All cells were cultured at 37ºC under 5% CO_2 in a high-humidity atmosphere.

Immunoblotting

Cells were lysed in RIPA buffer containing 1X protease inhibitor cocktail. Protein concentration was quantified using the BCA protein concentration assay kit (Pierce). Cell lysates were electrophoresed on SDS-polyacrylamide gels and transferred to a PVDF membrane (Millipore). Membranes were incubated with primary antibodies in TBST overnight at 4°C. The membranes were then incubated with HRP-conjugated secondary antibodies for 1 h at room temperature, and visualized using an ECL kit (Millipore). The antibodies used for immunoblotting were as follows:

MECP2 (#3456, CST), MECP2 (sc-137070, Santa cruz), ZEB1 (#3396), N-Cadherin (#13116), Vimentin (#5741), Slug (#9585), KLF4 (#4038), Sox2 (#3579)(CST), CD133 (ab19898), MMP14 (ab51074), GAPDH (ab8245)(Abcam).

Immunohistochemistry (IHC) analysis

Fresh 4-μm sections were cut from paraffin-embedded tissue samples. After the sections were baked (65ºC, 30 min), they were deparaffinized in xylene, then rehydrated in graded ethanol solutions. The sections were boiled with citrate buffer for 5 min for antigenic retrieval. The sections were incubated with a primary antibody overnight at 4°C. As a negative control, PBS was used to verify the antibody specificity. After washing, anti-rabbit or anti-mouse secondary antibodies (Zhongshan Biotech, Beijing, China) were used. The sections were incubated with DAB (3,3-diaminobenzidine), counterstained, dehydrated, and mounted in permanent mounting medium. IHC stained sections were reviewed and

scored independently by two superior pathologists. A final score was calculated by multiplying the proportion of positively stained tumor cells (0–100%) with the staining intensity (0,1,2,3).

RNA extraction and qRT-PCR

Total RNA from the cell lines was extracted using TRIzol Reagent (Invitrogen, USA) according to the manufacturer's instructions. cDNA was synthesized via reverse transcription (RT) according to the manufacturer's protocol (Takara, Dalian, China). PCR amplification was performed in triplicate using the SYBR Green PCR kit (Takara, Dalian, China). GAPDH was used as a control. The following primers were used:

GAPDH (forward: 5′-GTCTCCTCTGACTTCAACAGCG-3′; reverse: 5′-ACCACCCTGTTGCTGTAGCCAA-3′)

MECP2 (forward: 5′-TGAAGGCTGGACACGGAAGCTT-3′; reverse: 5′-CAGGGATGTGTCGCCTACCTTT-3′)

ZEB1 (forward: 5′-GGCATACACCTACTCAACTACGG-3′; reverse: 5′-TGGGCGGTGTAGAATCAGAGTC-3′)

Vimentin (forward: 5′-AGGCAAAGCAGGAGTCCACTGA-3′; reverse: 5′-ATCTGGCGTTCCAGGGACTCAT-3′)

ZEB2 (forward: 5′-AATGCACAGAGTGTGGCAAGGC-3′; reverse: 5′-CTGCTGATGTGCGAACTGTAGG-3′)

β-catenin (forward: 5′-CACAAGCAGAGTGCTGAAGGTG-3′; reverse: 5′-GATTCCTGAGAGTCCAAAGACAG-3′)

TCF3 (forward: 5′-CCAGACCAAACTGCTCATCCTG-3′; reverse: 5′-TCGCCGTTTCAAACAGGCTGCT-3′)

TCF4 (forward: 5′-GCCTCTTCACAGTAGTGCCATG-3′; reverse: 5′-GCTGGTTTGGAGGAAGGATAGC-3′)

Twist1 (forward: 5′-GCCAGGTACATCGACTTCCTCT-3′; reverse: 5′-TCCATCCTCCAGACCGAGAAGG-3′)

ZO-1 (forward: 5′--3′: GTCCAGAATCTCGGAAAAGTGCC-3′; reverse: 5′-CTTTCAGCGCACCATACCAACC-3′)

Co-immunoprecipitation (IP)

Cell lysates were incubated with specific antibodies overnight at 4°C, followed by precipitation with Protein A/G agarose beads (Santa Cruz, sc-2003) at 4°C for 1 h. Cell lysates from MECP2 stably-expressing HCT116 cells were subjected to co-IP with 5 μg SPI1 antibody (#2258, CST), followed by Western blot with MECP2 (3456, CST) and SPI1 (sc-365208, Santa Cruz).

Cell migration and invasion assays

A total of 1 × 105 cells/well were loaded into an insert with serum-free medium and allowed to adhere to a polycarbonate filter that was either pre-coated with 50 μl of Matrigel for the invasion assay or uncoated for the migration assay. The lower chambers were filled with DMEM or IMDM and 10% FBS. Cells on the upper surface of the filters were wiped out after 48 h. Cells that had migrated and invaded through the Matrigel were then fixed and stained with crystal violet. The membranes containing migrated and invaded cells were counted in five randomly selected microscopic views.

Wound healing assay

CRC cells were seeded into 6-well plates at a density of 1 × 105 cells/well in medium containing 10% FBS and cultured until ~80–90% confluence. The cells were scraped with a 10 μl pipette tip to generate

scratch wounds. The cells were washed twice with serum-free DMEM to remove cell debris. To record scratch wound closure, images were captured at 0, 48, and 96 h time points in the same position.

In vivo metastasis assays

HCT116 cells were washed with 1X PBS. For the intravenous injection, a total of 1 × 106 cells in 0.1 mL of DMEM were injected into the tail vein of 6-week-old male nude mice. To assess the degree of tumor formation in the lung, imaging of living mice was performed on an Inveon small-animal SPECT/CT imaging system equipped with an isoflurane 2% anesthesia system at six weeks post-injection. After eight weeks, animals were sacrificed and the lungs were harvested. Collected lung tissues were fixed in 10% buffered formalin solution overnight. Fixed tissues were stained with hematoxylin and eosin (H&E).

Cell sphere formation

A total of 500 cells were plated into 6-well ultralow attachment plates (3471, Corning) with serum-free medium containing 50 ng/mL epidermal growth factor (EGF; 236-EG-200, R&D), 20 ng/mL of basic fibroblast growth factor (bFGF; 233-FB-025, R&D), 1% N2 (17502048, Gibco), and 2% B27 (12587-010, Gibco). After 10 days, the number of spheres in each well with a diameter greater than 100 μm was counted under the microscope.

DNA extraction and bisulfite pyrosequencing analysis

Genomic DNA from fresh-frozen tissues and cells was isolated using MiniBEST Universal Genomic DNA Extraction Kit Ver.5.0 (TaKaRa), according to the manufacturer's instructions. An EpiTect Bisulfite Kit (QIAGEN, 59104) was applied to conduct the bisulfite modification of DNA (1–2 μg). PyroMark Assay Design Software 2.0 (Qiagen) was used to design the bisulfite pyrosequencing primers. The PyroMark Q96 System and software (Qiagen) were utilized for the sequencing reaction and methylation level quantification.

CCK8 assay

Cell proliferation was evaluated using the CCK8 assay (MCE, NJ, USA) according to the manufacturer’s instructions. The colon cancer cells were transfected with PLKO or shMeCP2, or transfected with Lenti-V2 or sgMeCP2, and plated into 96-well plates (2,000 cells per well) containing 100 μL media. Then, colon cancer cells were cultured for 0 h, 24 h, 48 h and 72 h and incubated with CCK-8 reagent at a final concentration of 10 μL/mL for 2 h at 37°C. The plate was mixed gently on an orbital shaker for 5 min before the absorbance was measured at 450 nm with a Multiskan FC microplate reader (ThermoFisher, USA). Each experiment was repeated three times.

Cell apoptosis

Cell apoptosis was assessed using FITC Annexin V Apoptosis Detection Kit with PI (BioLegend, CA, USA) according to the manufacturer’s instructions. After labelling, the cells were quantified on a BD Accuri™ C6 Flow Cytometer (BD Biosciences). The FlowJo software (Tree Star, Inc.) was used for the post-acquisition analysis of dot plots and histograms. A total of 50,000 events were acquired from each sample.

Cell cycle

Cells were collected 48 h after transfection, and fixed in 75% ethanol at 4°C overnight. The cells were then resuspended in 500 μL PBS and mixed with 50 μg/mL propidium iodide (PI, BioLegend, USA). After incubation for 30 min at 37°C in the dark, the stages of the cell cycle were analyzed using a flow cytometer.

Supplement figures

Fig. S1

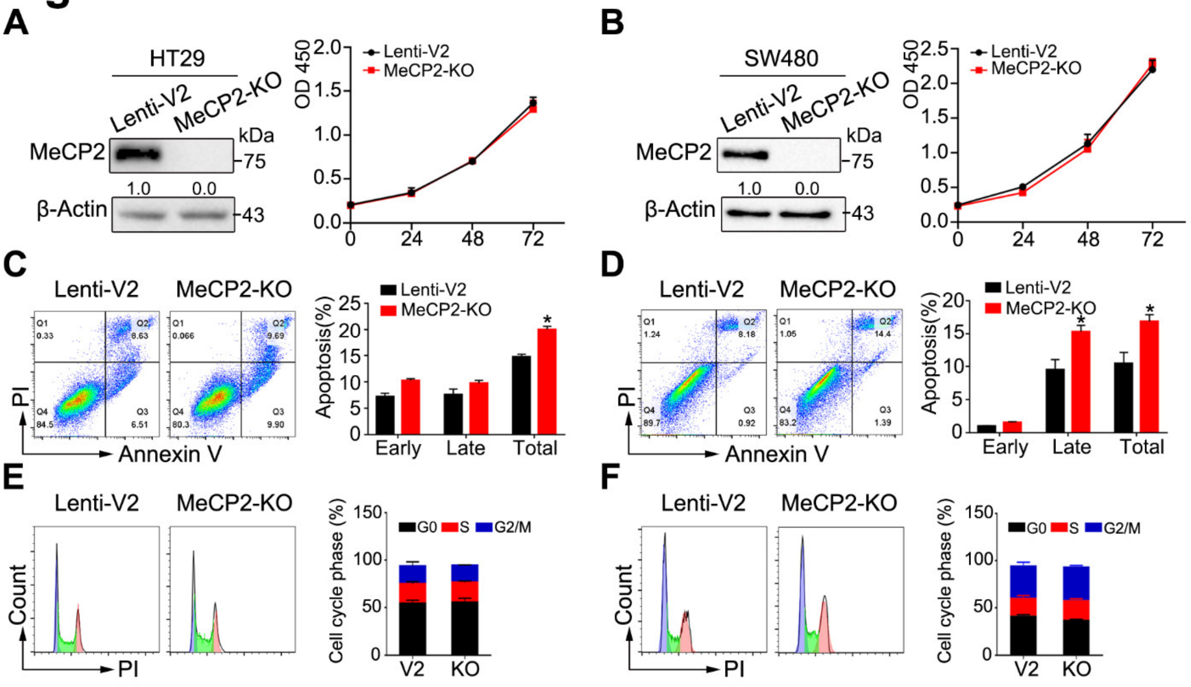

Figure S1 A sgRNA-mediated MeCP2 knockout determined by Western blot (left). MeCP2 abrogation did not influence cell viability in HT29 cells (right). **B** sgRNA-mediated MeCP2 knockout determined by Western blot (left). MeCP2 abrogation did not influence cell viability in SW480 cells (right). **C** MeCP2 knockdown did not affect apoptosis of HT29 cells. **D** MeCP2 knockout did not influence apoptosis in SW480 cells. **E** MeCP2 knockout in HT29 cells did not influence the cell cycle distribution. **F** MeCP2 knockout in SW480 cells did not affect cell cycle distribution

Fig. S2

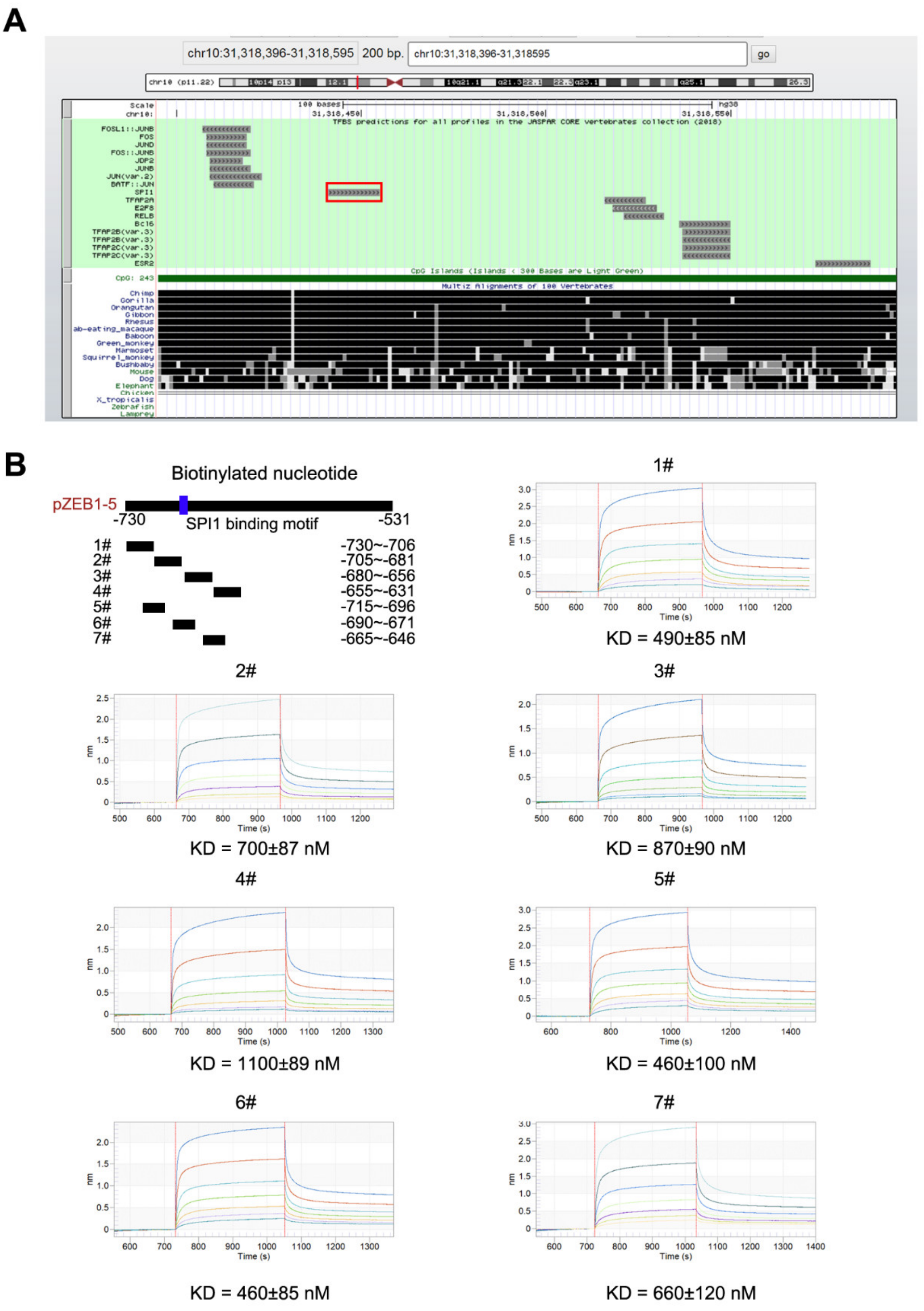

Figure S2 A Prediction of transcription factor binding to the pZEB1-5 promoter region using the UCSC genome browser. B Bio-Layer Interferometry (BLI) analysis of the binding of seven biotin-nucleotides to the minimal binding domain (MBD) of the MeCP2 protein

Fig. S3

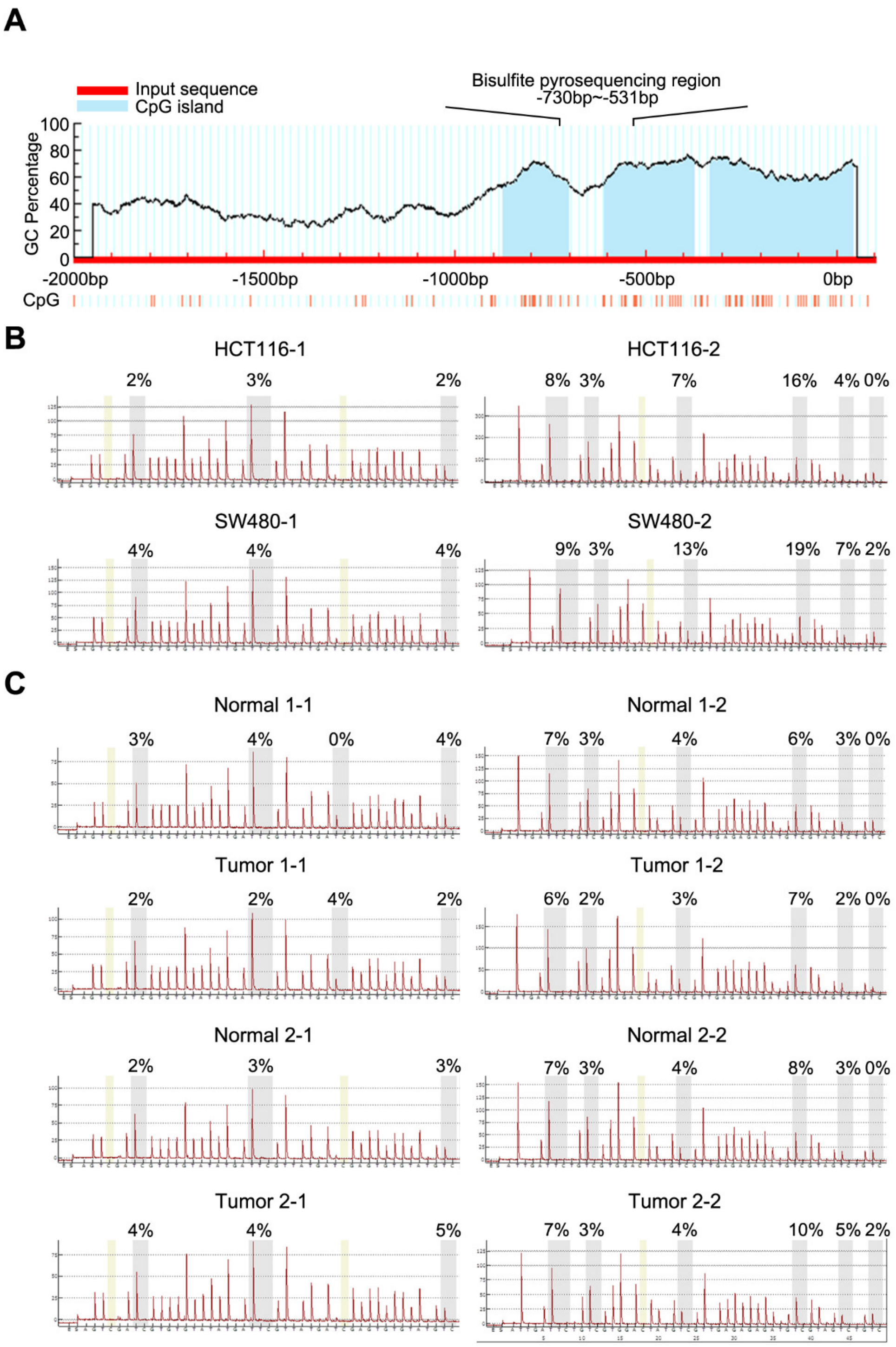

Figure S3 A Schematic of the CpG islands and bisulfite pyrosequencing region in the ZEB1 promoter. Input sequence: red region; CpG islands: blue region. B Bisulfite pyrosequencing analysis of the ZEB1

promoter region in HCT116 and SW480 cells. C Bisulfite pyrosequencing analysis of the ZEB1 promoter region in normal (n = 2) and CRC (n = 2) tissues

Fig. S4

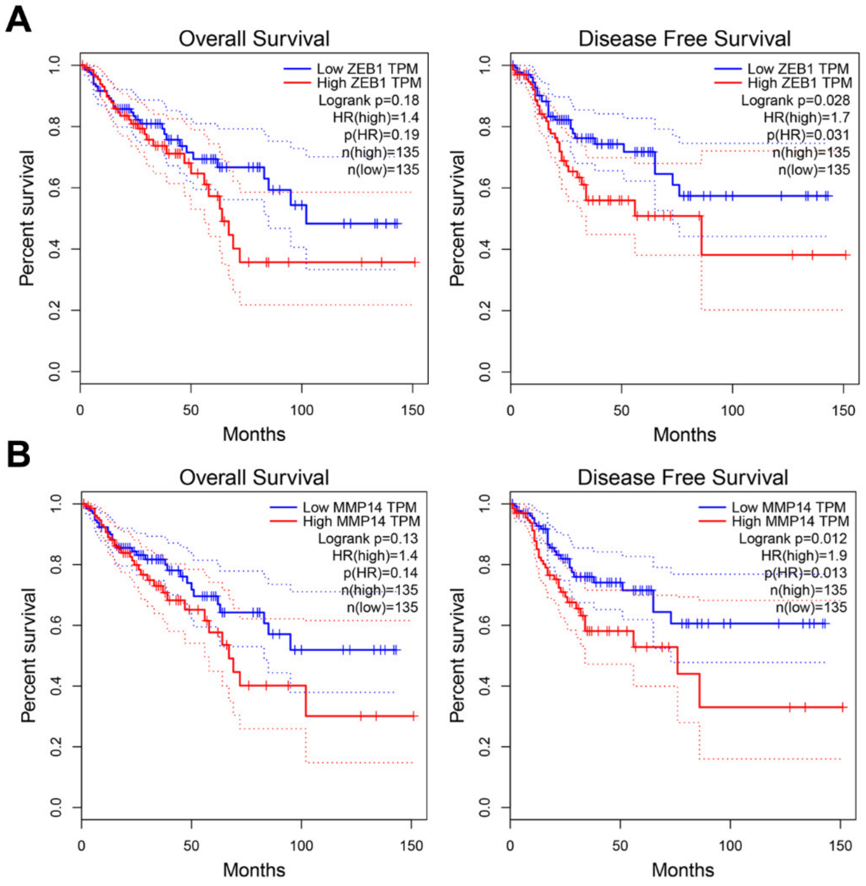

Figure S4 A Correlation between ZEB1 expression and survival in TCGA database for CRC, Kaplan–Meier analysis of overall survival time (left) and disease free survival time (right). B Correlation between MMp14 expression and survival in TCGA database for CRC, Kaplan–Meier analysis of overall survival time (left) and disease free survival time (right).
